# Uncovering the new landscape of leukoaraiosis through the circular RNA-miRNA-mRNA axis

**DOI:** 10.3389/fneur.2025.1603935

**Published:** 2025-11-04

**Authors:** Canmin Zhu, Chang Chang, Qiangjian Jin, Xi Xu, Weili Pang, Yang Fang, Ting Yang, Lanying Jin, Dili Wang

**Affiliations:** Department of Neurology, The First People’s Hospital of Jiangxia District, Wuhan, Hubei, China

**Keywords:** leukoaraiosis, miRNA, circRNA, interaction, mRNA

## Abstract

**Background:**

White matter disease arises from the loss of oligodendrocyte progenitor cells and is associated with adverse outcomes in leukoaraiosis (LA) patients. Although the regulatory roles of circ-RNA/miRNA/mRNA in brain disorders are well-documented, their impact and mechanisms in white matter injury (WMI) remain unclear. This study aimed to investigate the involvement of hsa_circ_0018401/miR-145-5p/AIFM1 axis in white matter injury.

**Method:**

Whole blood samples from LA patients underwent quantitative real-time polymerase chain reaction (qRT-PCR) to evaluate the expression levels of circular RNA and miRNA. Bioinformatics tools were employed to predict the downstream miRNA. Primary oligodendrocyte progenitor cells (OPCs) were isolated from rats. The downstream miRNA, miR-145-5p, of circ-0018401 in OPCs was confirmed through Luciferase gene assays and qRT-PCR. Luciferase gene assays were also used to explore the interaction between miR-145-5p and AIFM1 in OPCs and HEK-293T cells. Western blotting and qRT-PCR were utilized to analyze the expression levels of AIFM1 in OPCs overexpressing miR-145-5p.

**Results:**

Elevated levels of circ-0018401 were observed in the whole blood of patients with white matter injury. Overexpression of circ-0018401 resulted in decreased levels of miR-145-5p in OPCs. Luciferase gene assays and qPCR verified the binding of circ-0018401 with miR-145-5p in OPCs. The apoptotic gene AIFM1 was identified as a downstream target of miR-145-5p.

**Conclusion:**

circ-0018401 acts as a crucial biomarker in white matter injury by regulating the miR-145-5p/AIFM1 axis. This research opens up a new path for the development of therapeutic strategies for white matter injury.

## 1 Introduction

Leukoaraiosis (LA), also known as white matter lesions (WMLs), is a condition characterized by the degeneration and demyelination of nerve fibers in the brain, often accompanied by glial reactive hyperplasia (1). The prevention of oligodendrocyte progenitor cell death is crucial for myelin repair and the restoration of white matter integrity (2). Various factors contribute to the pathogenesis of LA, including hypertension, smoking history, diabetes, hyperlipidemia, hyperhomocysteinemia, and heart disease (3). The pathogenesis of cognitive dysfunction in LA remains unclear but is thought to be associated with several factors. Firstly, age-related white matter changes play a significant role as the brain’s white matter degrades with age, leading to volume reduction and structural alterations that can result in cognitive impairments (4). The pathology of LA is multifaceted, with blood flow hypoperfusion injury being a widely recognized mechanism. Additionally, damage to the blood–brain barrier and cerebral edema are important factors in LA (5). Several theories exist regarding the development of LA, with nerve circuit fiber connection interruptions being a potential key factor (6). Understanding the complex interplay of these mechanisms is essential for developing effective treatment strategies for LA and its associated cognitive dysfunction. Further research into the pathogenesis of LA is crucial for identifying novel therapeutic targets and interventions to mitigate the impact of this condition on cognitive function and brain health.

Circular RNA (circRNA) is prevalent in eukaryotic transcripts, particularly abundant in the nervous system. Known for its conservative, specific, and stable expression patterns, circRNA shows promise as a valuable diagnostic and therapeutic marker for various nervous system disorders. Notably, its expression abundance surpasses that of other RNA by up to tenfold, highlighting its significance in neurobiology (7). Advancements in bioinformatics have enhanced our understanding of the mechanisms and biological characteristics of circRNAs. Although many researchers have contributed their effort in unraveling these aspects, the regulatory impact of circRNAs on target gene expression remains largely unexplored. CircRNAs exhibit versatile functions; they can influence parental gene transcription and even encode proteins, explaining of complexity to their biological roles (8). Studies have revealed the widespread presence of circRNAs in nervous system tissues, implicating their involvement in various pathophysiological processes of neurological disorders. These processes include inflammation, oxidative stress, mitochondrial dysfunction, and cell apoptosis, underscoring the multifaceted roles of circRNAs in neurobiology (9). Consequently, the discovery of circRNAs has laid a solid theoretical foundation for understanding the pathogenesis of nervous system diseases, opening new avenues for therapeutic interventions and diagnostic strategies in this field.

Many reported has been shown that circular RNAs regulates neural function through various mechanisms. Firstly, circRNAs act as sponge molecules that absorb microRNAs (miRNAs), thereby modulating gene expression levels (9). Secondly, circRNAs can influence gene expression by regulating parental gene transcription or even encoding proteins. For instance, Ayers and Scerri (10) identified a specific circRNA with a ribosomal insertion site capable of encoding a 16 kD protein, demonstrating the protein-coding potential of circRNAs. Furthermore, circRNAs exhibit additional functions, such as binding to diverse miRNAs to disrupt downstream RNA targets (11). Studies have highlighted the significance of circRNAs in neurodegenerative diseases like Alzheimer’s disease (AD). For example, the depletion of ciRS-7 in the brain tissue of AD mouse models can elevate miRNA-7 levels, leading to the inhibition of miRNA-7 target UBE2A expression and influencing the progression of AD (12). These findings underscore the diverse roles of circRNAs and miRNA in neural regulation and their potential implications in neurodegenerative disorders.

MicroRNAs (miRNAs) represent a fundamental class of small single-stranded RNA molecules, typically 20–22 nucleotides in length, known for their ability to posttranscriptionally modulate gene expression by binding to the 3′ UTR region of target mRNAs (13). These miRNAs are pivotal regulators of essential processes in neurons, including proliferation, differentiation, apoptosis, and development (14), playing critical roles in various human disorders such as neurodegenerative and neuropsychiatric conditions (15). Remarkably, miRNAs exhibit stability in human bodily fluids (16), with their expression levels in plasma holding promise as prognostic biomarkers for various diseases (17). Despite their significance, there remains a scarcity of studies investigating the epigenetic factors involved in regulating the transcription of risk genes associated with leukoaraiosis (LA). Specifically, research exploring miRNA profiles and the pathogenetic role of miRNAs in LA is notably lacking, presenting an opportunity for further exploration and understanding in this area (18).

Bioinformatics tools have revolutionized our understanding of the molecular targets and underlying biological mechanisms of diseases, providing a systematic, accurate, and effective approach to elucidating the theoretical basis for disease occurrence (19). In the context of leukoaraiosis (LA), a condition whose pathogenesis remains unclear, research on circular RNAs (circRNAs) involved in cerebrovascular injury, neural aging, and protein synthesis is still in its nascent stages, with many aspects awaiting comprehensive explanation. Researchers are increasingly turning to the construction of circRNA-miRNA-mRNA networks using bioinformatics methods to explore the intricate biological mechanisms underlying LA and other neurological disorders. PCR validation studies have demonstrated dysregulated expressions of specific circRNAs, such as down-regulated circRNA_016800 and up-regulated circRNA_002170, indicating their potential roles in modulating disease-associated mRNA through circRNA-miRNA-mRNA interactions, thus contributing to the pathophysiology of persistent brain diseases (20). Delving into circRNA/miRNA/mRNA co-expression networks at key nodes is deemed crucial for unraveling the mechanisms involved in the onset and progression of conditions like ischemic stroke (21). Atherosclerosis, a chronic inflammatory disease intricately linked to cardiovascular and cerebrovascular conditions, underscores the importance of investigating the interplay between circRNAs and their competitive mRNAs in disease development (22). Furthermore, in-depth gene network component analyses can unveil the molecular intricacies and identify key central genes contributing to the complex mechanisms underlying various brain diseases. This comprehensive approach sheds light on the interconnected pathways and molecular players involved in the pathogenesis of intricate neurological conditions, offering valuable insights for potential therapeutic interventions and targeted treatments.

In this research, we delved into the identification of circRNA biomarkers in whole blood samples from patients with white matter hyperintensities (WHI) utilizing qRT-PCR analysis. Additionally, we embarked on a comprehensive exploration of the potential roles of dysregulated miRNAs in the pathogenesis of WHI, employing a combination of molecular biology techniques and bioinformatics methodologies. Our investigation involved the screening of differentially expressed circRNAs in the whole blood of WHI patients, followed by an in-depth analysis of their mechanisms of action. Through the construction and prediction of molecular biological networks, we aimed to uncover how these circRNAs are intricately involved in regulating downstream factors and influencing the reverse transcription process. By adopting a molecular biology perspective, our study sought to shed light on the pathogenesis of WHI, offering novel insights and strategies for early intervention and treatment approaches in the realm of white matter hyperintensities.

## 2 Materials and methods

### 2.1 Participants and sample collection

Our study adhered to ethical research guidelines involving human participants and obtained approval from the Clinical Research Ethics Committee of the Jiangxia District First People’s Hospital before commencing the research. Written consent for participation was secured from patients or their legal guardians. The study encompassed five healthy individuals and five patients aged 38–79, admitted to Hospital between June 2022 and December 2024. Clinical samples were acquired from patients diagnosed with leukoaraiosis (LA), confirmed through magnetic resonance imaging (MRI) revealing white matter lesions. Participants were excluded if they: (A) declined participation, (B) exhibited conditions like mental disorders, aphasia, or sensory impairments affecting cognitive assessment, and (C) had severe concurrent illnesses such as neurodegenerative disorders, tumors, or immunological diseases. Blood samples were collected from each individual on the 14th day post-LA onset and stored at −80 °C for further analysis (23).

### 2.2 Animals

The animal research carried out in this study adhered to the guidelines outlined in the Helsinki Declaration, was authorized by the National Institutes of Health regulations, and received approval from the Laboratory Animal Ethical Committee of the Clinical Research Ethics Committee of the Jiangxia District First People’s Hospital. Healthy SD rat pups, regardless of sex, aged 3 days postnatal, were procured from the Jiangxia District First People’s Hospital and accommodated in the institution’s animal facility. Primary oligodendrocyte precursor cells (OPCs) were extracted from rat pups on postnatal day 3, while the remaining rats were raised until they reached adulthood at 2 months old, weighing between 200–220 g (five rats in each group).

### 2.3 Experimental groups

Following Zhu’s et al. (2) method, briefly, the rats were randomly divided into sham group and WHI group (five rats in each group). The intraventricular hemorrhage model was established after anesthesia in the WHI rat model, and was established by injecting autologous blood around the left periventricular area. The same procedure was conducted in sham group but were not injected with autologous blood. After sham or WHI treatment, rats were euthanized to obtain brain tissue for further analysis.

### 2.4 Primary oligodendrocyte precursor cells and cell culture

Following Zhu’s et al. (2) method with slight adjustments, primary oligodendrocyte precursor cells (OPCs) were isolated from the brains of SD rat pups by sham or WHI (five rats in each group). The brain tissue was dissected, then meninges and blood vessels were removed, and the cells were obtained after centrifugation with trypsin. Next, cells were filtered through a 70 μm mesh and cultured for 12 to 14 days. Microglia were eliminated by agitation, followed by shaking at 200 rpm to obtain OPCs from the astrocyte layer. OPCs were then cultured in serum-free basal medium DMEM for 3 to 5 days. HEK-293T cells were cultured in EMEM medium supplemented with 10% fetal bovine serum, 100 U/mL penicillin, and 0.1 mg/mL streptomycin.

### 2.5 Cell transfection

The overexpression vector for circ_0018401 (circ_0018401 ov), miR-145-5p mimics (miR-145-5p), along with their respective controls, were procured from a source in Ruibo Limited, China. The oligodendrocyte precursor cells (OPCs) were grown in culture plates until they reached around 80% confluence. Subsequently, they were transfected with the designated plasmids using Lipofectamine 3000 (Invitrogen, Carlsbad, CA, United States), following the manufacturer’s instructions and in accordance with the methodology outlined by Zhu’s et al. (2).

### 2.6 RNA extraction and reverse transcription-quantitative polymerase chain reaction

Total RNA was isolated from whole blood samples of patients utilizing Trizol LS reagent (Invitrogen, United States) in strict adherence to the manufacturer’s instructions. The extraction protocol closely followed Qiao’s et al. (23) methodology. Similarly, total RNA was extracted from brain tissues from rat or oligodendrocyte precursor cells (OPCs) using TRIzol. For circRNAs and mRNAs, reverse transcription employed the PrimeScript RT master mix to obtain RNA, GAPDH as internal control. Reverse-transcribed miRNAs were obtained by using the Bulge-Loop miRNA PCR starter kit with unique stem-loop primers (normalized to U6). PCR reactions were conducted utilizing SYBR Premix Ex Taq II (TaKaRa, Tokyo, Japan) and a 7500 Real-Time PCR System (Applied Biosystems, CA, United States). The qPCR analysis primers are listed in Table 1. Data was analyzed by using the 2^−ΔΔCT^ method to calculate the relative expression levels of gene.

**Table 1:** Primer.

miRNA	Sequence	References
miR-127-5p	Forward: 5′-GCCGAGCTGAAGCTCAGAGG	(44)
	Reverse: CTCAACTGGTGTCGTGGA	
miR-377	Forward: CGCGCTCCTATATGATGCCT	(45)
	Reverse: GTCGTATCCAGTGCAGGGTCCGAGGTATTCGCACTGGATACGACGAAGAA	
miR-421	Forward: ATCAACAGACATTAATTGGGCGC	(24)
	Reverse: GCGAGCACAGAATTAATACGAC	
miR-532-3p	Forward: CCTCCCACACCCAAGGCTTGCA	(46)
	Reverse: CAAGCCTTGGGTGTGGGAGGTT	
miR-145-5p	Forward: GTCCAGTTTTCCCAGGAATC	(25)
	Reverse: AGAACAGTATTTCCAGGAAT	
U6	Forward: CTCGCTTCGGCAGCACATATACT	(25)
	Reverse: ACGCTTCACGAATTTGCGTGTC	
AIFM1	Forward: TCTGGACACTGGCAAACATC	(25)
	Reverse: GCTTTCCCCAGAAAGACACA	
GAPDH	Forward: GCAAGTTCAACGGCACAG	(25)
	Reverse: GCCAGTAGACTCCACGACAT	
ciRS-7	Forward: TACCCAGTCTTCCATCAACTGG	(47)
	Reverse: ACACAGGTGCCATCGGAAAC	
circ_101396	Forward: AAAGGTCCACTTCGTATGCTG	(27)
	Reverse: ACTCTGTCATTGGAGCAACTGTAT	
circ_102533	Forward: GCTGCCAAAAGCATAACCAA	(27)
	Reverse: CCCCTTTTCTGCTAAATGAACTCT	
circ_102470	Forward: CCTAAATTTCACGACACCAG	(27)
	Reverse: ATTCAGATTGCTCAAGGTAACT	
circ-DLGAP4	Forward: ACGGCTACTGGTTCCTAAAGC	(28)
	Reverse: GGGGTCTTCTTATACGCCACT	
circ_0018401	Forward: AGCCAAGATGACCAGAA	(23)
	Reverse: AATGACTAAATATGCCCAC	

### 2.7 Luciferase gene reporter

The method was followed by Hong’s et al. (24), with minor modification. We engineered wild-type and mutant 3′-untranslated regions (UTRs) for the anticipated AIFM1 or circ_0018401, synthesized these distinct fragment sequences, and subsequently integrated them into the luciferase vector.

Verification of all vectors was conducted through sequencing. Following this, either wild-type or mutant plasmids were co-transfected with miR-145-5p mimics or mimic NC into OPCs or HEK293T cells in 24-well plates. Firefly and Renilla luciferase activities were assessed utilizing the Dual Luciferase Assay System (E2920, Promega).

### 2.8 Western blotting

The methodology closely followed Zhou’s et al. (25) procedure with minor adjustments. Total protein was extracted from oligodendrocyte precursor cells (OPCs) utilizing RIPA buffer. Subsequently, the extracted protein was separated through SDS-PAGE and transferred onto PVDF membranes. Following blocking, the membranes were subjected to an overnight incubation with primary antibodies, which included AIFM1 (anti-AIFM1, 1:1,000, Solarbio, K003425P) and the GAPDH control (1:2,500, Ab9485, Abcam, Cambridge, United Kingdom). The following day, the membranes were washed with a washing buffer and then exposed to horseradish peroxidase (HRP)-conjugated secondary antibodies (1:1,000, Ab97110, Abcam, Cambridge, United Kingdom) for 1 h at room temperature. Finally, protein bands were visualized and detected using chemiluminescence.

### 2.9 Statistical analysis

The results were presented as the mean ± standard deviation of three independent experiments. Statistical analyses were conducted using GraphPad Prism 7.0 (GraphPad Software Inc., San Diego, United States). Student’s *t*-test was employed for comparing two groups, while one-way ANOVA was utilized for comparisons among multiple groups. Statistical significance was denoted as follows: ^*^p < 0.05, ^**^p < 0.01, ^***^p < 0.001, ^****^p < 0.0001, and ns, no significant.

To predict downstream miRNAs from circ-0018401, the CircInteractome web tool was employed, and the results were visualized using Cytoscape software. Further investigation into the predicted candidate targets of miRNA was carried out using Targetscan, an online database for miRNA target prediction and functional annotations, also visualized using Cytoscape software. The STRING database^1^ was utilized for analyzing and visualizing lists of proteins or genes, creating GO enrichment, and protein–protein interaction networks for target genes.

## 3 Results

### 3.1 Analysis of the level of expression of circRNAs in LA patients

**Figure 1:**
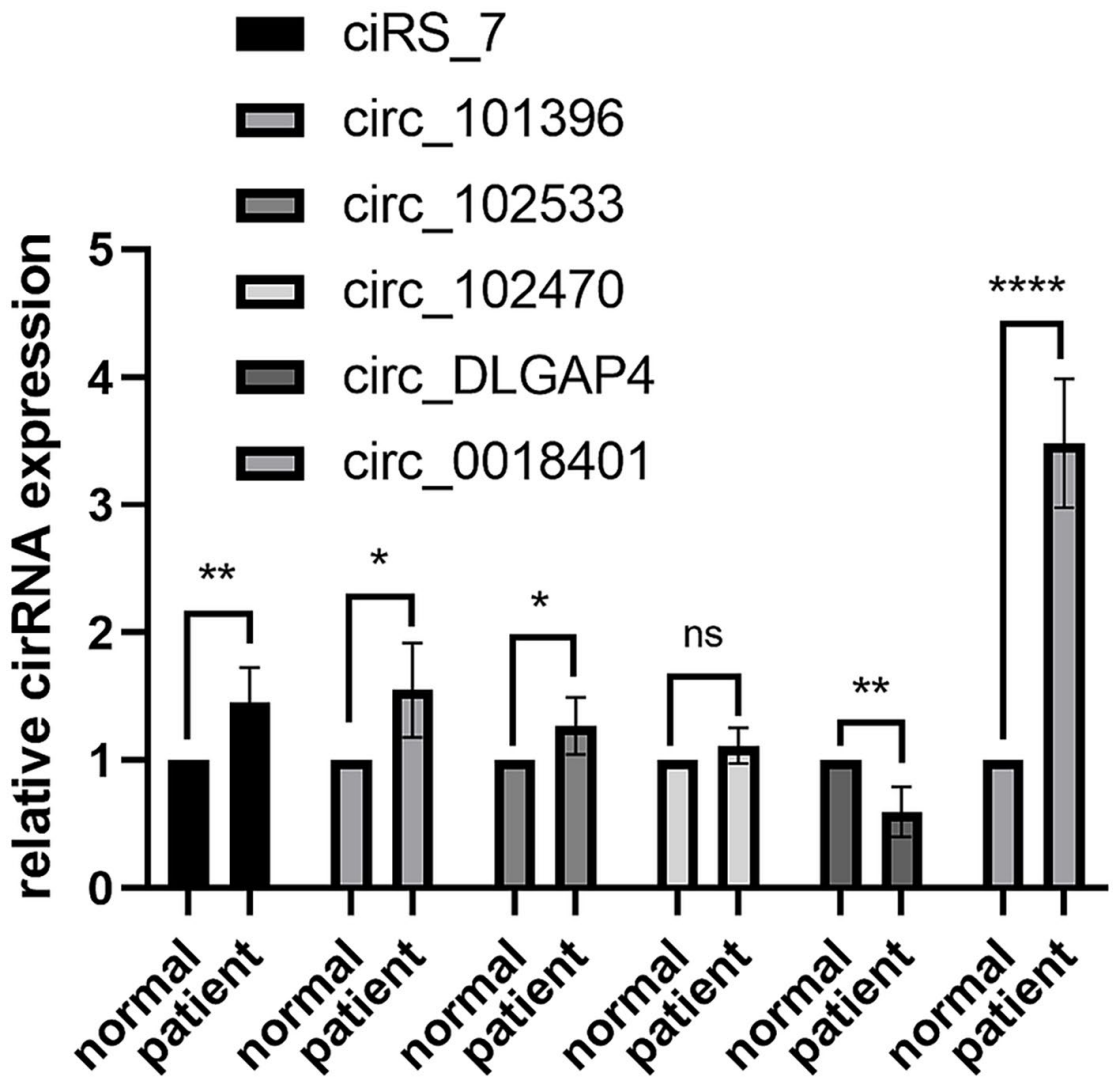
Expression of circRNAs in normal participants (*n* = 5) and LA (*n* = 5) patient groups.

Six circRNAs ciRS-7 (26), circ_101396 (27), circ_102533 (27), circ_102470 (27), circ DLGAP4 (28), circ_0018401 (23) were selected, which were reported they are related to brain disease. Reverse transcription-quantitative polymerase chain reaction (RT-qPCR) data revealed that circ_0018401 was significantly up-regulated (*p* < 0.0001) in patient blood sample (*n* = 5, Figure 1). Previous studies have also demonstrated that hsa_circ_0018401 was related to white matter injury (23), suggesting that hsa_circ_0018401 might be a potential biomarker of WHI, so this circRNA was selected for following study.

### 3.2 miRNA prediction and validation

**Figure 2:**
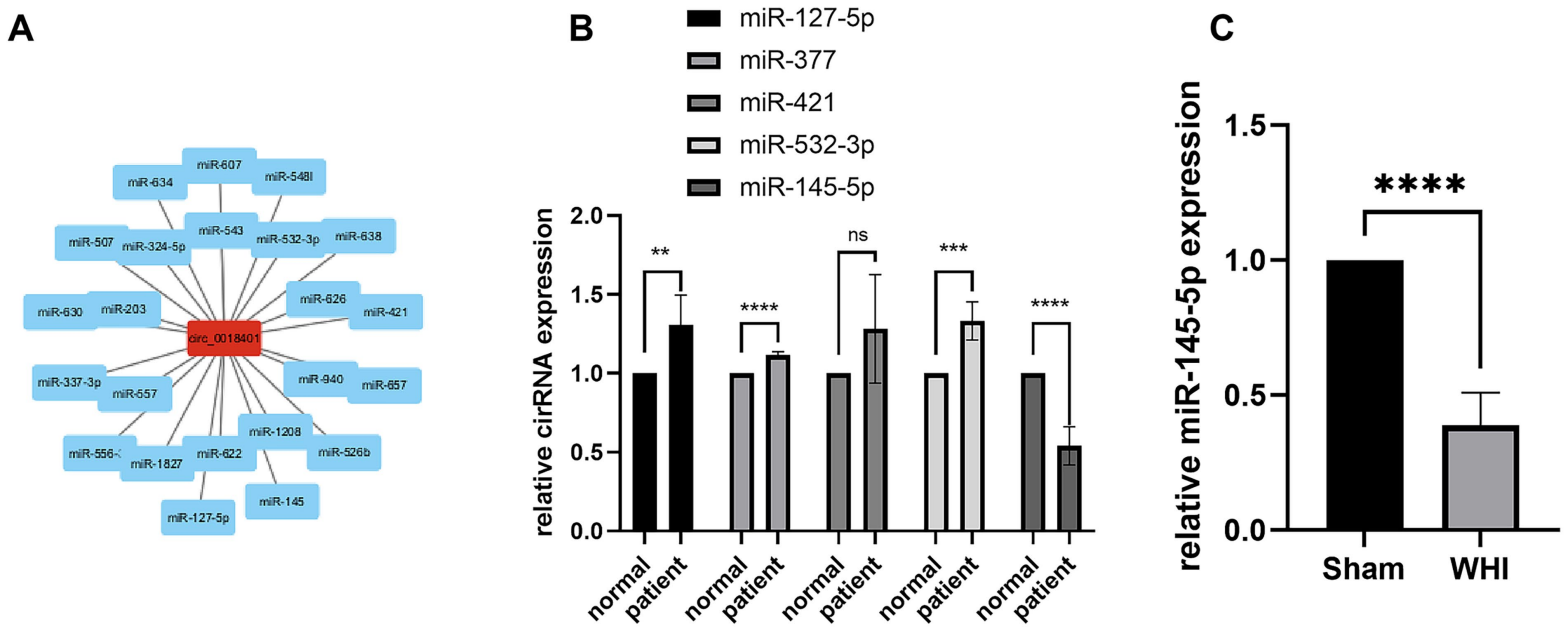
miRNA prediction and validation. **(A)** Network of circ_0018401 and predicted miRNAs. **(B)** Expression of circRNAs in normal participants (*n* = 5) and LA (*n* = 5) patient groups. **(C)** Expression of miR-145-5p in WHI rat with comparison of sham group (*n* = 5) and WHI rat (*n* = 5) groups.

The regulatory circRNA-miRNA network was predicted using Circletome, identifying 23 miRNAs. The interaction networks of circ_0018401 were visualized with Cytoscape software (Figure 2A). RT-qPCR analysis indicated that miR-145-5p was significantly downregulated (*p* < 0.01) in blood samples from patients, while other miRNAs did not show significant differences (*n* = 5, Figure 2B). Subsequently, we examined miR-145-5p expression in rat brain tissue. The RT-qPCR results revealed that miR-145-5p was downregulated (*p* < 0.01) in the brain tissue of WMI rats compared to sham rats (*n* = 5, Figure 2C). Previous studies have also established a network between miR-145-5p and brain diseases, demonstrating that miR-145-5p targets MMP2 to help protect against brain injury (29), suggesting that miR-145-5p might be a potential biomarker of LA.

### 3.3 Circ_0018401 binds with miR-145-5p in OPCs

**Figure 3:**
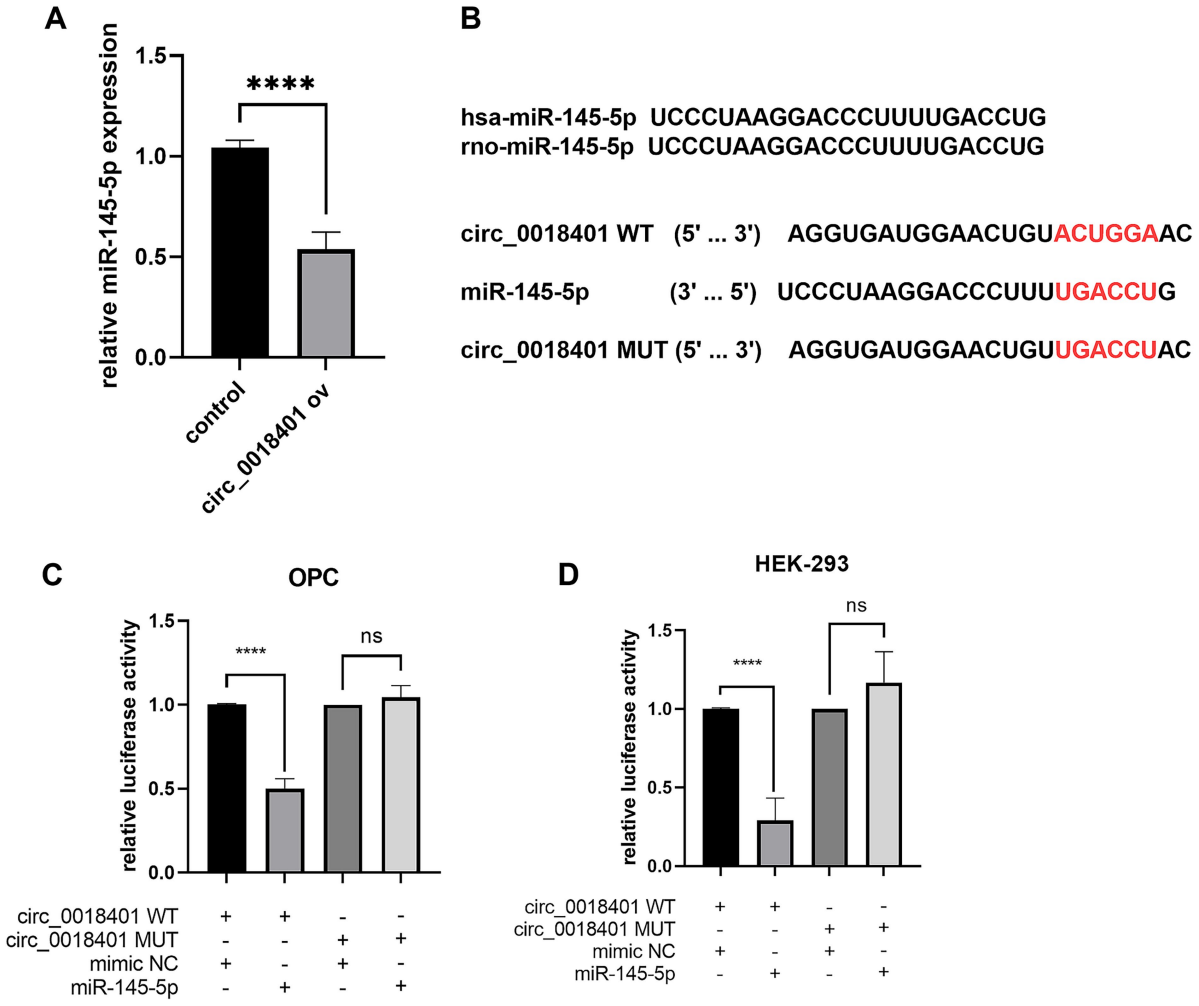
Circ_0018401 binds with miR-145-5p in OPC. **(A)** Binding sequence between miR-145-5p and circ_0018401. **(B)** RT-qPCR measured miR-145-5p level in circ_0018401 over expression OPC. Luciferase reporter assay detected binding relation between circ_0018401 and miR-140-3p in OPCs **(C)** and HEK-293T cells **(D)**.

After selecting miRNAs based on database predictions, we hypothesized miR-145-5p as a downstream target of circ_0018401. Expression levels of miR-145-5p were significantly downregulated (*p* < 0.0001) in oligodendrocyte precursor cells (OPCs) overexpressing circ_0018401 via transfection, compared to those with an empty vector (*n* = 5, Figure 3A). Bioinformatic analysis revealed that miR-145-5p has a conserved sequence across species and contains a binding site on circ_0018401 (Figure 3B). This suggests that circ_0018401 negatively regulates miR-145-5p in OPCs.

In both OPCs and HEK-293T cells, co-transfection with wild-type or mutant circ_0018401 luciferase reporters, along with miR-145-5p or mimic NC, was performed. As shown in Figures 3C,D, miR-145-5p significantly reduced (*p* < 0.0001) the luciferase activity of the wild-type circ_0018401 reporter, but had no effect on the luciferase activity of the mutant circ_0018401 reporter.

### 3.4 MiR-145-5p targets AIFM1 *in silico*

**Figure 4:**
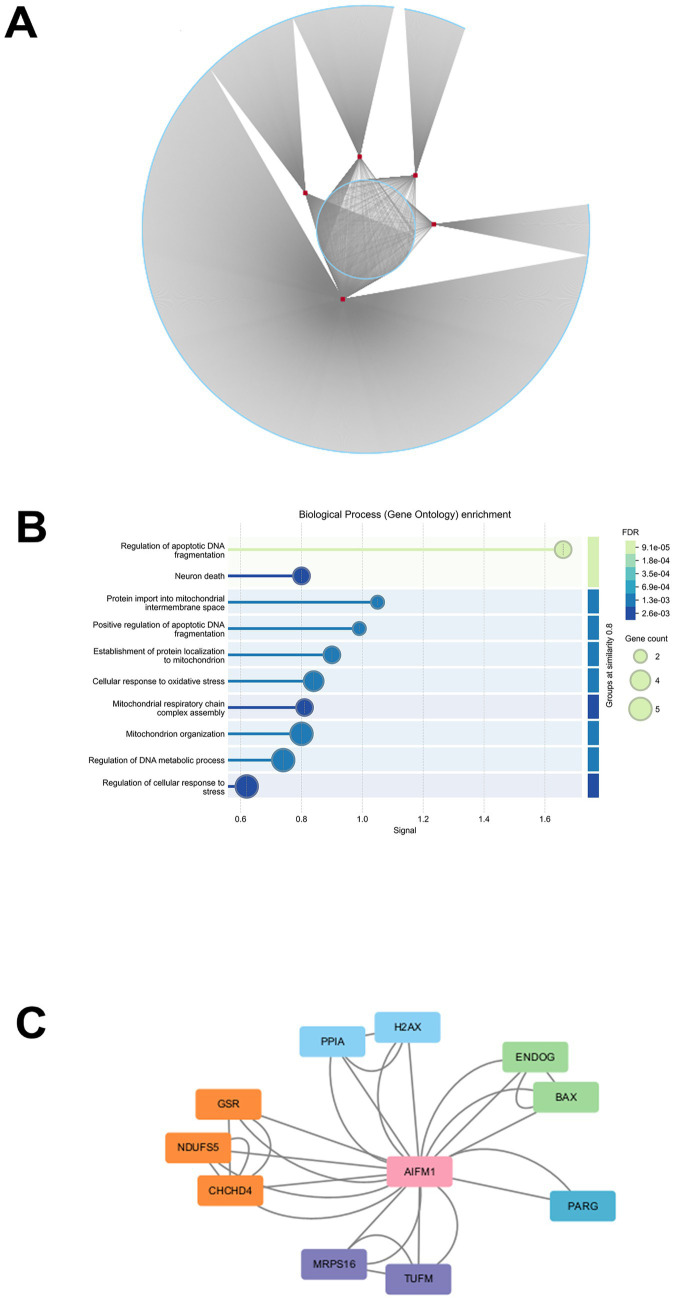
Network of miRNA and predicted mRNA establishment. **(A)** Overall regulatory miRNA-mRNA network. The red and blue circles represent miRNAs and mRNAs, respectively. The continuous and dotted lines connecting the nodes indicate direct and indirect regulation, respectively. **(B)** GO enrichment of AIFM1. **(C)** Protein–protein interaction (PPI) network of AIFM1.

We established a miRNA and predicted target gene network using TargetScan, selecting five miRNAs (miR-127-5p, miR-377, miR-421, miR-532-3p, miR-145-5p) to construct the network in Cytoscape (Figure 4A). As mentioned above, miR-145-5p was selected to explore its bio-function this study. It is associated with the apoptotic gene AIFM1, according to Targetscan database prediction. This candidate mRNA, AIFM1, was analyzed using the STRING database, revealing links to apoptotic DNA regulation, neuronal death, and cellular oxidative stress (Figure 4B). Additionally, the protein–protein interaction (PPI) network indicated that AIFM1 is related to ENDOG and BAX (Figure 4C). Previous studies have shown that AIFM1 is a downstream target of miR-145-5p and is involved in apoptosis (25). Furthermore, apoptosis of OPC is essential in white matter injury (2). Therefore, we focused on miR-145-5p to investigate its relationship with AIFM1.

### 3.5 MiR-145-5p targets AIFM1 in OPCs

**Figure 5:**
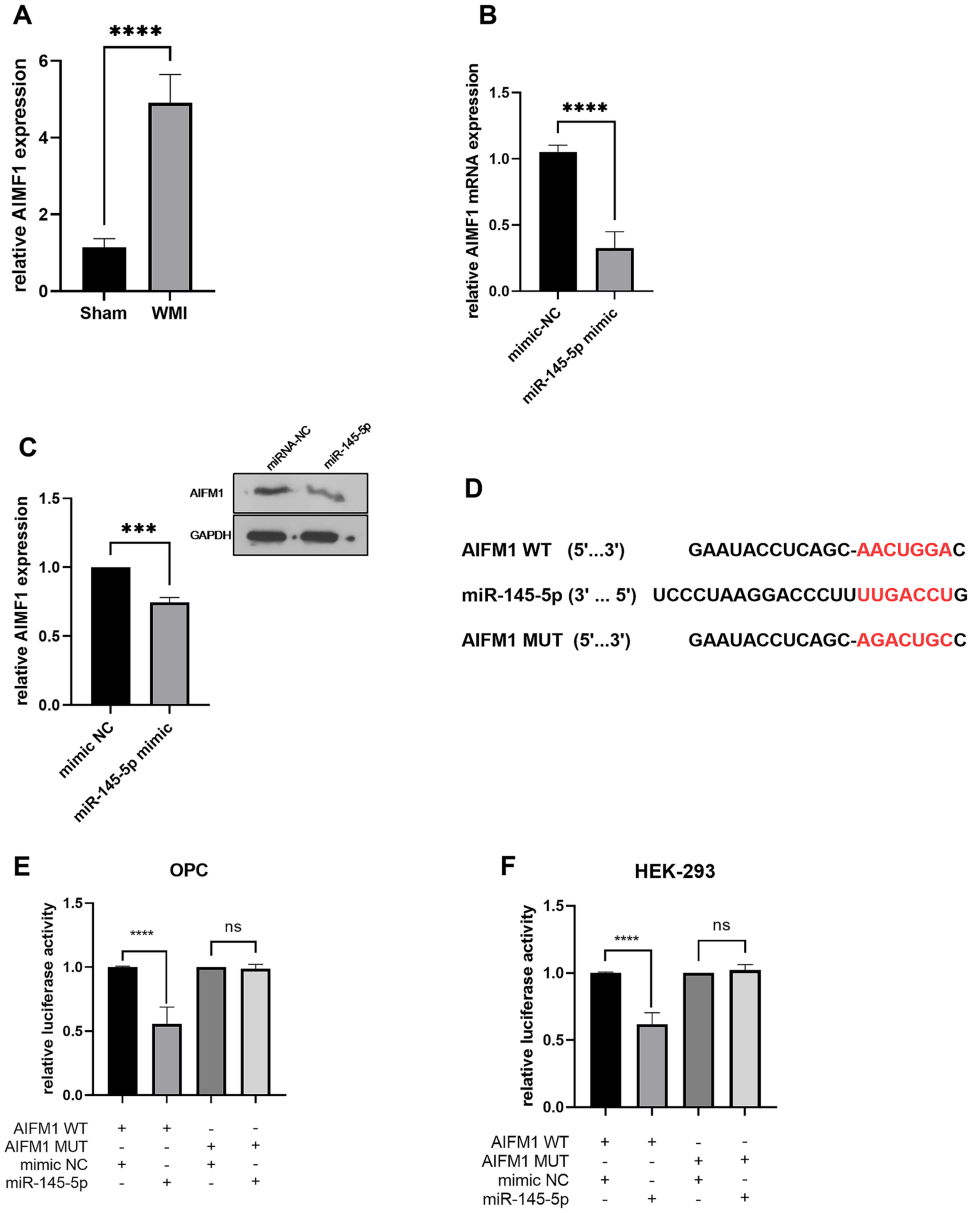
AIFM1 is the target gene of miR-145-5p in OPCs. **(A)** AIFM1 mRNA level in rat brain tissues in WMI or sham group detected by RT-qPCR. **(B)** AIFM1 mRNA level in OPCs transfected with miR-145-5p mimics detected by RT-qPCR. **(C)** Relative AIFM1 protein expression level in OPCs transfected with miR-145-5p mimics. **(D)** Binding sequence of miR-145-5p and AIFM1 3′ UTR. Binding relation between AIFM1 and miR-145-5p in OPCs **(E)** or HEK-293T cells **(F)** detected by luciferase assay.

RT-qPCR data indicated that AIFM1 was significantly upregulated (*p* < 0.0001) in the brain tissue of WMI rats compared to sham rats (*n* = 5, Figure 5A). Following the miR-145-5p mimic transfection in oligodendrocytes, AIFM1 levels were downregulated (*p* < 0.0001), suggesting that miR-145-5p negatively regulates AIFM1 expression in these cells (*n* = 5, Figure 5B). Western blot analysis further confirmed these results, showing a reduced (*p* < 0.001) level of AIFM1 when miR-145-5p was elevated in oligodendrocytes (Figure 5C).

We examined the binding sequence of miR-145-5p on the AIFM1 3′ UTR using bioinformatics and explored the relationship between miR-145-5p and AIFM1 in OPCs and HEK-293T cells through luciferase reporter assays in (Figure 5D). OPCs and HEK-293T cells were co-transfected with wild-type or mutant AIFM1 luciferase reporters along with miR-145-5p mimics or mimic NC. As shown in Figures 5E,F, miR-145-5p significantly decreased (*p* < 0.0001) the luciferase activity of the wild-type AIFM1 reporter, while it had no effect on the luciferase activity of the mutant AIFM1 reporter.

## 4 Discussion

Leukoaraiosis, a pathological manifestation in the brain’s white matter, has traditionally been attributed to perfusion irregularities within the arterioles (30). WMI stands out as the most prevalent form of brain injury, significantly contributing to severe neurological complications (31). In experimental animals, hematologic edema subsequent to intracerebral hemorrhage (ICH) predominantly impacts the cerebral white matter (32). Identified as an imaging marker of cerebral small vessel disease (CSVD), leukoaraiosis (LA) is pathologically characterized by pale myelin, demyelination, oligodendrocyte apoptosis, and vacuole formation (33). Understanding these associations is crucial for advancing research in neurology and developing targeted interventions to address the complexities of these conditions.

Circular RNAs (circRNAs) are a group of exceptionally stable and evolutionarily conserved molecules that are prominently expressed in brain related symptom (34). Recent research has shed light on the significance of circRNAs in brain-related conditions. For instance, in Alzheimer’s disease, ciRS-7 up-regulation can lead to change level of miRNA-7. This up-regulation deficiency in ciRS-7 results in reduced “sponging” effects, subsequently down-regulating several mRNA targets relevant to Alzheimer’s disease, including the ubiquitin conjugase protein (26). Studies have also pointed towards the potential of circRNAs as biomarkers for various neurological conditions. For example, has_circ_102533 has been proposed as a promising biomarker for diagnosing leukoaraiosis (27). Additionally, the interconnected expression levels of circMyt1l/rno-let-7d-5p/BDNF have been associated with periventricular white matter damage (35). In the realm of ischemic stroke, circular RNA DLGAP4 has been identified for its role in ameliorating the condition by regulating endothelial-mesenchymal transition linked to blood–brain barrier integrity (28). Another circRNA, circ_0018401, has been implicated as a potential biomarker related to the onset and progression of periventricular white matter damage (23). Despite the growing understanding of circRNAs in neurological disorders, the specific mechanisms by which circ_0018401 contributes to white matter injury (WMI) remain unclear. Our study delved into the impact of circ_0018401 on WMI. Significantly elevated levels of circ_0018401 were observed in the blood of patients with WMI compared to the control group. These findings hint at a pivotal role of circ_0018401 in WMI development in leukoaraiosis, suggesting that targeting circ_0018401 could be a promising therapeutic avenue for addressing WMI.

MicroRNAs (miRNAs) have emerged as pivotal players in oligodendrocyte development and cerebral injuries like WMI (36). Circular RNAs (circRNAs) have been recognized for their sponges effect for miRNAs, thereby modulating their expression levels. These circRNAs have been demonstrated to impact a wide range of biological processes by selectively binding to specific miRNAs. Through this interaction, they actively participate in many cellular activities, modulating gene expression, signaling pathways, and cellular functions essential for the proper functioning of the nervous system. Their intricate regulatory roles highlight their significance in orchestrating complex molecular networks within neural tissues (37). Competing endogenous RNAs (ceRNAs) are transcripts that post-transcriptionally modulate each other by competing for shared miRNAs (38). For instance, circ-AGTPBP1 has been reported to negatively regulate miR-140-3p and exhibit dysregulated expression levels in white matter injury (2). To delve into this concept further, we employed bioinformatics tools to identify potential miRNAs that could interact with circ_0018401. Using the circinteractome, we predicted the network of downstream miRNAs associated with circ_0018401. Our analysis pointed towards miR-145-5p, which was markedly downregulated in the brain tissue of leukoaraiosis (LA) patients and WHI rats. This discovery positioned miR-145-5p as a promising candidate for further exploration, leading us to select it for subsequent investigations in our study.

Oligodendrocyte progenitor cells (OPCs) situated in both gray and white matter regions are recognized as a vital source for generating oligodendrocytes (39). In lesions associated with leukoaraiosis (LA), demyelination is often accompanied by the loss of cells, particularly oligodendrocytes (40). Through our analysis, we discovered a binding sequence of miR-145-5p on circ_0018401, indicating a direct interaction between these molecules in OPCs. Employing bioinformatics tools, we identified AIFM1 as a downstream target of miR-145-5p (Figure 4A), a relationship that has not been previously explored in the context of WMI. In this sutdy, AIFM1 related apoptosis and neuron death. AIFM1, short for apoptosis-inducing factor mitochondrion-associated 1, is known to trigger caspase-dependent apoptosis and inhibit cell growth in various cell types (41). The well-documented role of AIFM1 within mitochondria has garnered significant attention for its involvement in apoptosis (42). Previous studies have established AIFM1 as a downstream gene of miR-145-5p (25). Moreover, OPC apoptosis is a critical factor in white matter injury (2). Strategies aimed at mitigating white matter injury must consider approaches that suppress the ongoing process of cell apoptosis to preserve the integrity of the white matter (43). Our study findings indicated an upregulation of AIFM1 expression in the brain tissue of rats with white matter injury (WMI). Additionally, we observed a direct interaction between miR-145-5p and the 3′ UTR of AIFM1 in OPCs, underscoring the significant role of circ_0018401 in mediating OPC phenotypic effects.

Nevertheless, it is crucial to recognize the limitations inherent in this study. Firstly, our focus was solely on OPCs to explore the biological functionality and mechanism of circ_0018401. Future investigations should delve into the impact of circ_0018401 on various other cell types and tissues. Secondly, the PPI network indicated it involve the apoptotic signaling pathway apoptotic biomarker such as BAX, so the study’s confinement to the assessment of the apoptotic effect of the circ_0018401/miR-145-5p/AIFM1 axis *in vivo* may need a fully investigation. Despite these constraints, our research implies that circ_0018401 exerts a substantial influence on the regulation of white matter injury by modulating the miR-145-5p/AIFM1 axis. These discoveries present a promising avenue for the development of therapeutic interventions targeting white matter injury.

## 5 Conclusion

In summary, our study demonstrates that circ_0018401 interacts with miR-145-5p in OPCs. Furthermore, we identified that miR-145-5p targets the apoptotic gene AIFM1, leading to the establishment of the circ_0018401/miR-145-5p/AIFM1 axis. These findings contribute to elucidating the molecular biological mechanism of white matter injury (WMI) and offer potential therapeutic target biomarkers for leukoaraiosis (LA).

## Data Availability

The original contributions presented in the study are included in the article/Supplementary material, further inquiries can be directed to the corresponding author.

## Footnotes

^1^ https://string-db.org/
