# Supplementary figures and images for: Uncovering the new landscape of leukoaraiosis through the circular RNA-miRNA-mRNA axis
Source: Front Neurol. 2025 Nov 4;16:1603935. doi: 10.3389/fneur.2025.1603935 (PMC12623190; doi:10.3389/fneur.2025.1603935)

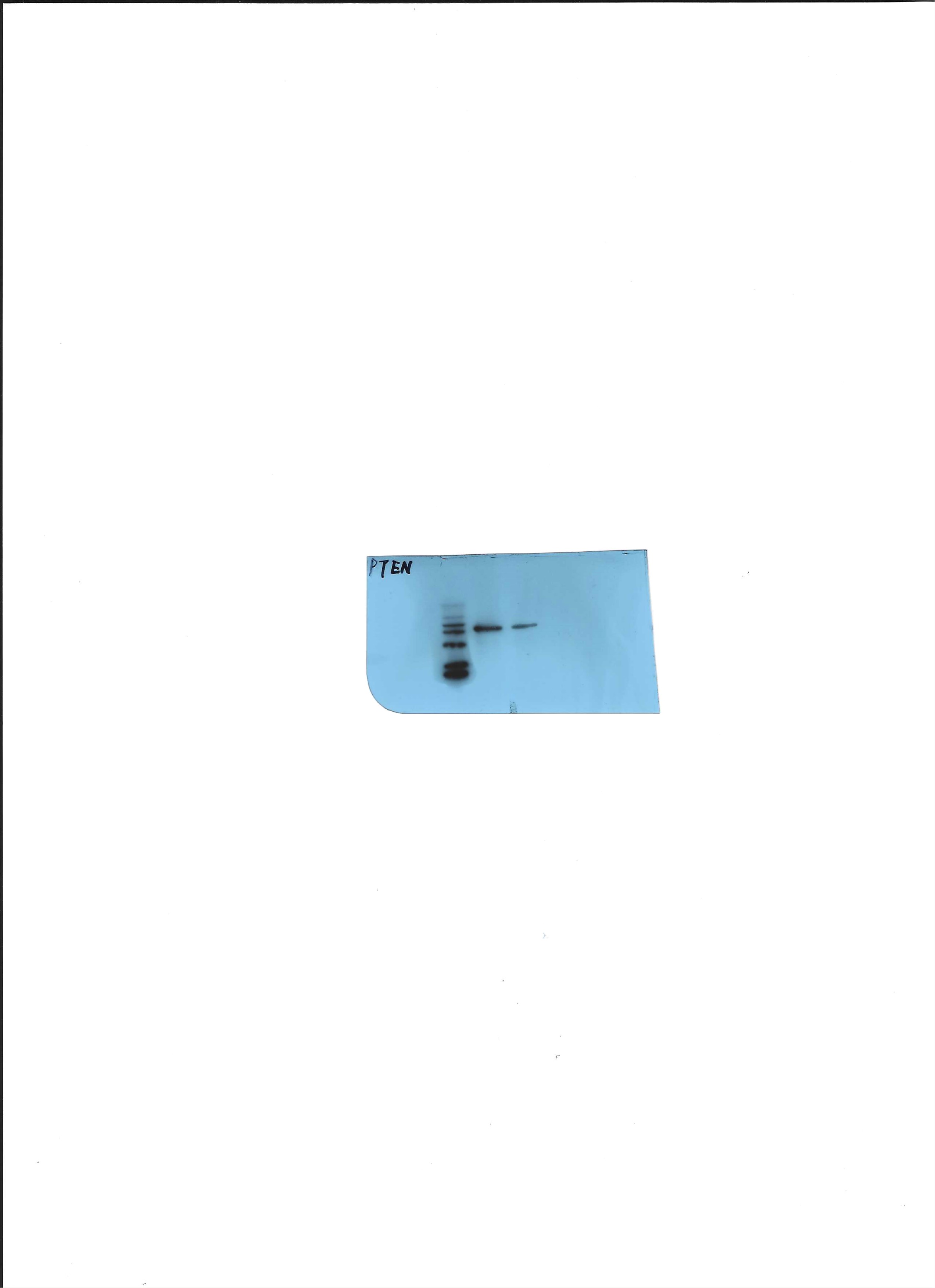

Supplement: Supplementary file 1 [file Image_1.jpeg]
